# Supplementary material for: Analysis of the Circulating Tumor Cell Capture Ability of a Slit Filter-Based Method in Comparison to a Selection-Free Method in Multiple Cancer Types
Source: Int J Mol Sci. 2020 Nov 27;21(23):9031. doi: 10.3390/ijms21239031 (PMC7730626; doi:10.3390/ijms21239031)
Supplement: Supplementary file 1 [file ijms-21-09031-s001.zip › Figure S1/Figure S1legend.pdf]

Figure S1. CTC diameter analysis of the two methods about the three prostate cancer cell samples (the 3 PCa) or the other samples (33 samples excluding the 3 PCa). The 3 PCa that showed a much higher cell count by the RareCyte method than the CTC-FIND method were analyzed separately from the analysis of the other samples. The black and white bars indicate the CTCs obtained by the CTC-FIND method and the RareCyte method, respectively. The X-axis indicates the cell diameter classifications. The graph shows an X-axis with 2.6-μm intervals. The blue and red line indicate a connecting line of the CTC-FIND method and the RareCyte method, respectively. (a) The size distribution of CTCs collected by the CTC-FIND method or the RareCyte method from the 3 PCa. (b) The CTC diameter distribution and the number of CTCs in each range among the CTCs collected by each method from the 33 blood samples excluding the 3 PCa.
