# Supplementary figures and images for: Analysis of the Circulating Tumor Cell Capture Ability of a Slit Filter-Based Method in Comparison to a Selection-Free Method in Multiple Cancer Types
Source: Int J Mol Sci. 2020 Nov 27;21(23):9031. doi: 10.3390/ijms21239031 (PMC7730626; doi:10.3390/ijms21239031)

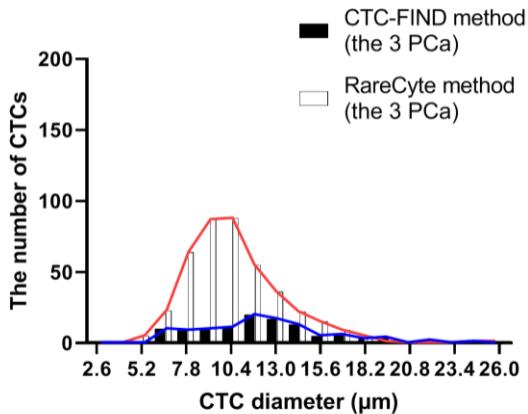

(a)

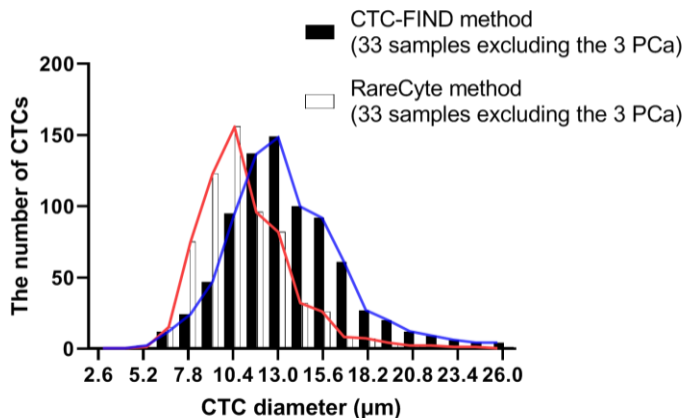

(b)

Supplement: Supplementary file 1 [file ijms-21-09031-s001.zip › Figure S1/Figure S1.pdf]
